# Production and Semi-Automated Processing of ^89^Zr Using a Commercially Available TRASIS MiniAiO Module

**DOI:** 10.3390/molecules25112626

**Published:** 2020-06-05

**Authors:** Vijay Gaja, Jacqueline Cawthray, Clarence R. Geyer, Humphrey Fonge

**Affiliations:** 1 Department of Medical Imaging, University of Saskatchewan, College of Medicine, Saskatoon, SK S7N 0W8, Canada; vijay.gaja@lightsource.ca; 2 Canadian Light Source, Saskatoon, SK S7N 2V3, Canada; 3 Saskatchewan Centre for Cyclotron Sciences, Saskatoon, SK S7N 5C4, Canada; jacqueline.cawthray@fedorukcentre.ca; 4 Department of Pathology and Laboratory Medicine, University of Saskatchewan, College of Medicine, Saskatoon, SK S7N 5E5, Canada; clg595@mail.usask.ca; 5 Department of Medical Imaging, Royal University Hospital, Saskatoon, SK S7N 0W8, Canada

**Keywords:** Zirconium-89, automation, radiolabelling

## Abstract

The increased interest in ^89^Zr-labelled immunoPET imaging probes for use in preclinical and clinical studies has led to a rising demand for the isotope. The highly penetrating 511 and 909 keV photons emitted by ^89^Zr deliver an undesirably high radiation dose, which makes it difficult to produce large amounts manually. Additionally, there is a growing demand for Good Manufacturing Practices (GMP)-grade radionuclides for clinical applications. In this study, we have adopted the commercially available TRASIS mini AllinOne (miniAiO) automated synthesis unit to achieve efficient and reproducible batches of ^89^Zr. This automated module is used for the target dissolution and separation of ^89^Zr from the yttrium target material. The ^89^Zr is eluted with a very small volume of oxalic acid (1.5 mL) directly over the sterile filter into the final vial. Using this sophisticated automated purification method, we obtained satisfactory amount of ^89^Zr in high radionuclidic and radiochemical purities in excess of 99.99%. The specific activity of three production batches were calculated and was found to be in the range of 1351–2323 MBq/µmol. ICP-MS analysis of final solutions showed impurity levels always below 1 ppm.

## 1. Introduction

Zirconium-89 (^89^Zr), a positron-emitting isotope, has emerged as an attractive radionuclide in the development of pre-clinical and clinical radiopharmaceuticals for positron-emission tomography (PET) imaging. This is due to the favourable physical characteristics of the isotope which decays via positron emission (*T*_1/2_: 78.4 h, β^+^ 22.7%, E_β+max_ = 901 keV; average E_β+max_ = 396 keV) [1,2] and electron capture (EC 77%, Eγ = 909 keV) to the stable yttrium-89 (^89^Y). The longer half-life matches the need for large biomolecules such as antibodies, antibody fragments and nanoparticles which require prolonged circulation time in order to reach optimal target accumulation [3,4]. Furthermore, the β^+^ branching ratio, average β^+^ energy and short positron range (R^ave^ = 1.23 mm) provide high spatial resolution and image quality of the ^89^Zr PET probes [5]. 

The combination of antibody-based targeting vectors and PET-based imaging, known as immuno-PET, is rapidly becoming a powerful tool for highly selective imaging agents [6,7]. Radiolabeling of the targeting vector requires a stable radiometal chelate that can be readily conjugated to an antibody or other proteins. At present, the bifunctional derivative of desferrioxamine (DFO), *p*-isothiocyanatobenzyl-desferoxamine (*p-SCN-Bz*-DFO) is considered the gold-standard and with facile reaction chemistry has had widespread application for preclinical research and clinical trials [8]. However, recently there have been some concerns surrounding the in vivo instability of the ^89^Zr-DFO complex, presumably due to the unsaturated coordination sphere, which has led to the generation of new chelators with enhanced stability of the resulting ^89^Zr complex [9,10,11].

The most common ^89^Zr production method is via the ^89^Y(p,n)^89^Zr transmutation reaction. This route, employing ^89^Y targets that is in 100% natural abundance and commercially available, is readily accessible by most medical cyclotrons. In general, the target is irradiated with incident proton beam energies of 13–16 MeV. At these low energies, there is a trade-off between radionuclidic purity and efficiency of the process owing to the competing reactions to produce ^88^Zr via the ^89^Y(p, 2n)^88^Zr reaction and ^88^Y via the ^89^Y(p, pn)^88^Y reaction at threshold energy of 13.3 MeV. The average maximum cross section value for ^88^Zr is 786.9 ± 1.5 at 22.98 MeV and for ^88^Y is 298.0 ± 5.7 mb at 28.30 MeV [1]. The production of ^88^Zr and ^88^Y is minimized at 12.8 MeV due to small cross sections and threshold energy. Following dissolution of the irradiated target in concentrated HCl the isolation and purification of ^89^Zr proceeds via cation exchange chromatography using hydroxamate-modified resin [1,12]. Zirconium-89 is selectively eluted from the column using 1 M oxalic acid. With optimized conditions, this standardized method of separation can achieve high recovery, radionuclidic purity and effective specific activity (ESA) [1].

The increased interest in ^89^Zr-immuno-PET imaging probes for use in preclinical and clinical studies has led to a rising demand for ^89^Zr. Although ^89^Zr is commercially available, the lengthy transportation time and high costs are a barrier for preclinical and routine clinical applications of ^89^Zr immuno-PET. Furthermore, the highly penetrating 511 and 909 keV photons emitted by ^89^Zr deliver an undesirably high radiation dose, which makes it difficult to produce large amounts manually. So far, the processing of ^89^Zr from the target has mainly been achieved manually or has been based on semi-automated systems. These have not been widely adopted because they are based on in-house developed systems instead of commercially available equipment [13,14,15]. Moreover, regulatory demands are increasing and consequently the application of ^89^Zr in clinical studies is becoming challenging. To maintain the high quality of the ^89^Zr, a reliable process with quality control and good manufacturing practice-compliant automated module in the production is required. Additionally, there are several advantages of using automation in radiochemistry, such as lower radiation exposure to the production personnel, high reproducibility, more precise control of the parameters and ease of handling. Mostly, automated modules are placed inside dedicated lead shielded hot cells to protect the operator and environment from radiation exposure. Commercially available automated modules can be classified into two types, namely, those that consist of fixed tubing in which fluid flows are regulated by inert gas, and those that consist of sterile disposable cassettes where fluid flow is regulated by a syringe pump. The main disadvantage of using fixed tubing is bacterial contamination in the product if a validated cleaning method is not implemented. Usually this tubing is used for multiple productions and needs to be cleaned after each synthesis. Also, a single system cannot be used for multiple radioisotopes or for the production of other radiopharmaceuticals. These issues can be solved by using sterile, single-use cassettes manufactured in current Good Manufacturing Practices (cGMP)-compliant clean rooms.

The focus of this present work is the implementation of the semi-automated purification of ^89^Zr using a commercially available automated synthesis unit that could fulfil regulatory requirements.

## 2. Materials and Methods

### 2.1. General

A commercially available sterile, single-use cassettes (produced according to GMP) used for ^68^Ga-cationic prepurification (catalog number 10886) were purchased from TRASIS and adapted for the ^89^Zr purification. PEEK tubing and PEEK connectors were purchased separately from IDEX Health & Science, LLC (Middleboro, MA, USA) and sanitized with hydrogen peroxide solution and dried using nitrogen gas. A customized glass target dissolution vial with a vented glass cover that also allowed the insertion of PEEK tubing for the addition and removal of solutions into the vial and a aluminium heating block for the vial were manufactured at the University of Saskatchewan (Saskatoon, SK, Canada). All commercially obtained chemicals were of the highest available purity grade and were used without further purification. Hydrochloric acid (99.999% trace metal basis), water (Omni *Trace* Ultra™), oxalic acid (99.999% trace metals basis), acetonitrile (99.999% trace metal basis), *N*-(3-dimethylaminopropyl)-*N*¢-ethyl carbodiimide hydrochloride (99.9%), hydroxylamine hydrochloride (99.999% trace metal basis), 2,3,5,6- tetrafluorophenol (97%), diethylenetriaminepentaacetic acid (99%), 1.5 mL Eppendorf^®^ Safe-Lock microcentrifuge tubes, and empty reversible SPE tubes (0.5 mL) were purchased from Sigma Aldrich (Oakville, ON, Canada). Methanol (semiconductor grade) was purchased from Fisher Scientific (Ottawa, ON, Canada). *p-SCN-Bz*-DFO was purchased from Macrocyclics, Inc. (Dallas, TX, USA). PEEK tubing 1/16” OD × .040” ID, flangeless nut PEEK, Short, 1/4-28 flat-bottom, for 1/16” OD, flangeless ferrule tefzel, (ETFE), 1/4-28 flat-bottom, Luer-adapter assembly 1/4-28 female–male PEEK, and Luer-adapter 1/4-28 female to female Luer PEEK, were obtained from IDEX Health & Science, LLC (Middleboro, MA, USA). Sep-Pak Accell Plus CM light cartridges containing weak cation-resin were purchased from Waters (Milford, MA, USA). A tube rotator was purchased from VWR International (Mississauga, ON, Canada). Instant thin-layer chromatography paper (iTLC) was purchased from Agilent Technology (SantaClara, CA, USA). The radio-TLC plates were scanned using a Bioscan AR-2000 radio-TLC scanner (Washington, DC, USA). Radioactivity was measured using a Capintec CRC-55tr dose calibrator (Florham Park, NJ, USA) or a 1470 Wallac Wizard automated gamma counter (Perkin Elmer, Ramsey MN, USA).

### 2.2. Cyclotron Irradiation

Yttrium-89-coated niobium coins were purchased from Advanced Cyclotron Systems Inc. (ACSI, Richmond BC, Canada). The niobium target body had a diameter of 24 mm and a thickness of 1 mm. The yttrium was adhered on the frontal face of niobium target body with a diameter of 10 mm and approximately 200 µm thickness. Aluminium degrader coins (99.0% 24 mm with 0.80 mm thickness) were purchased from Goodfellow (Coraopolis, PA, USA). 

The target body along with 0.80 mm-thick aluminium degrader was mounted on TR-24 cyclotron (ACSI, Richmond, BC, Canada) at the Saskatchewan Centre for Cyclotron Sciences (SCCS) and bombarded for 2 h with the initial proton energy of 17.8 MeV and 40 µA current calculated as ≈ 12.8 MeV degraded beam energy at the target to produce ^89^Zr via the ^nat^Y(p, n)^89^Zr reaction. During irradiation, the target was cooled on the frontal side by helium gas and on the back side by chilled water. After irradiation, the target was left on the target station for 2–3 h to allow for the decay of short-lived isotopes, in particular ^88m^Zr (*t_1/2_* = 4.16 m). The target was released into a lead pig using target release valve located outside the cyclotron vault. The lead pig containing the irradiated target was manually retrieved from the vault and transported to the hot cells on a shielded cart. 

### 2.3. Automation Adopted for ^89^Zr Separation and Purification

**Figure 1:**
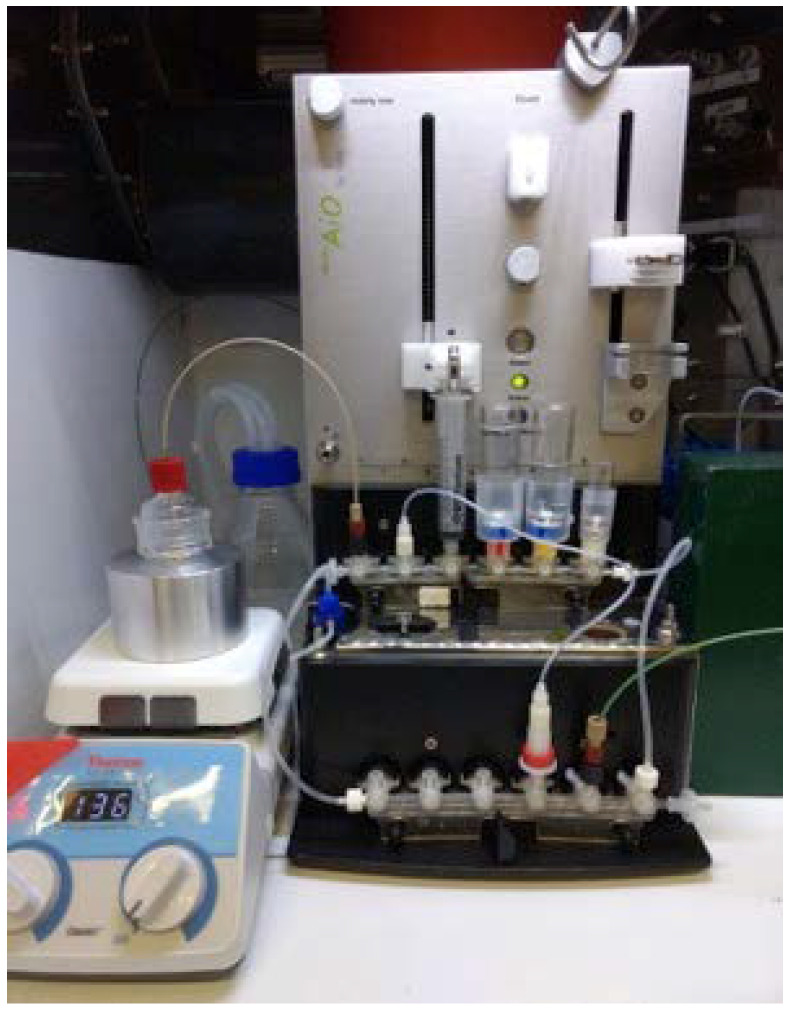
TRASIS mini AlliOne (miniAiO) setup for purification of zirconium-89.

A mini AlliOne (miniAiO) cassette-based automatic synthesis unit (ASU) from TRASIS (Ans, Belgium), with dimensions 21.5W × 41.2H × 40.8D cm, was used for the automated separation and purification of ^89^Zr (Figure 1). The miniAiO consists of 12 rotary actuators and 2 linear actuators for operating syringes. All liquids were transferred through PEEK tubing. All tube connections were assembled using non-metallic connectors to reduce exposure to the metals which may affect radionuclidic purity and specific activity. Many of the liquid connection pieces were flangeless fittings designed for high pressure fluidic connection and ferrules are manufactured from tefzel™ (ETFE). The fluidic path was controlled using the built-in three-way valve actuators. All liquids and hydroxamate resin cartridge were preloaded onto the cassette prior to starting the sequence on the miniAiO.

The miniAiO ASU was located within a lead hot cell and remotely controlled through a graphical user interface (PC). A sequence was created on the TRASIS software using a graphical user interface which distributes commands and controls to the automation unit. The operator can initiate a pre-programmed ^89^Zr target dissolution and purification process. Radioactive detectors are built-in at different locations within the ASU to monitor and record the location of the radioactivity. The radioactivity in the dissolution vial, hydroxamate cartridge and final vial was monitored. 

### 2.4. Target Dissolution and Purification of ^89^Zr

#### TRASIS MiniAiO Preparation

**Figure 2:**
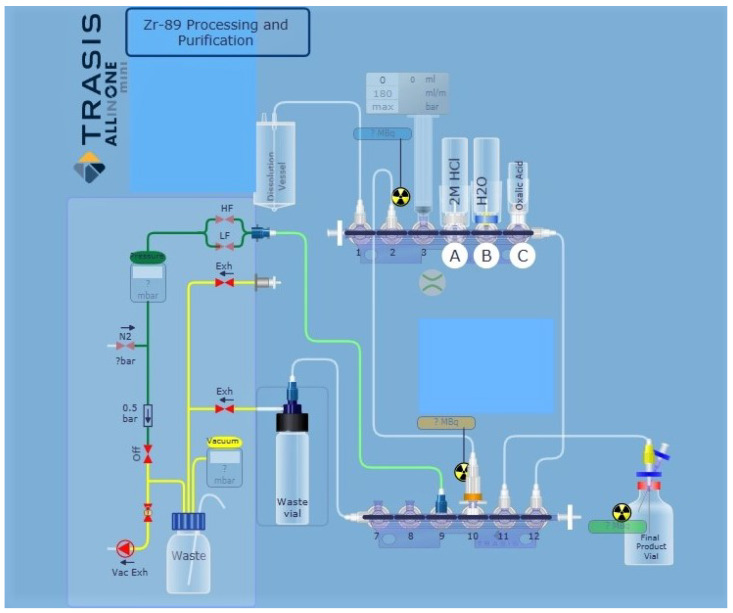
TRASIS miniAiO layout for automated purification and isolation of ^89^Zr from irradiated yttrium coin. Valve 1 to dissolution vial; valve 3, 10 mL syringe; valve 4 (**A**) 2 M HCl (20 mL); valve 5 (**B**) water (10 mL); valve 6 (**C**) 1 M oxalic acid (1.5 mL); valve 10 hydroxamate resin; valve 11 to product vial.

Automation of the process for separation and purification of ^89^Zr, based on chemistry previously reported in the literature, was performed using a miniAiO ASU in a hot cell [1,12,16]. The miniAiO is a commonly used radiochemistry module for clinical GMP-grade radiopharmaceutical production. The ASU is designed for use with disposable kits and allows processing with full audit trail functionality for GMP production runs. Liquid transport was achieved using a syringe pump and transfer of liquids was controlled by three-way stopcock valves. For each liquid transfer step, the production protocols were adequately adapted and optimized. In brief, the following general steps were implemented in the production of ^89^Zr. The hydroxamate resin (100 mg) was packed into an empty reversible SPE tube (0.5 mL). The hydroxamate resin was preconditioned with acetonitrile (MeCN) (8 mL, trace metal grade), water (15 mL, trace metal grade) and 2.0 M HCl (2 mL) and installed on the cassette (valve #10) of the miniAiO ASU (Figure 2). The following vials were installed on the cassette: 2 M HCl (20 mL) in a glass vial with septa installed at cassette valve #4, water (10 mL) in a glass vial with septa installed at cassette valve #5, 1 M oxalic acid (1.5 mL) in a glass vial with septa installed at cassette valve #6, a waste vial (40 mL) connected to cassette valve #7 and a [^89^Zr]zirconium oxalate product vial (5 mL) connected to cassette valve #11. 

The irradiated target was transferred to the custom-made dissolution vial and the vial was placed inside the aluminium block heater on a hot plate that had been pre-heated to 80 °C. After placing the vented glass cover with attached PEEK tubing on the dissolution vial, the hot cell was closed, and the purification sequence was initiated using the program interface for the miniAiO. Hydrochloric acid (2.0 M, 4 mL) was drawn into the syringe and pushed into the dissolution vial to dissolve the target. The target solution was heated at 80 °C for 20 min, and after cooling for 20 min, was passed through the hydroxamate resin using the syringe actuator at 1 mL/min flowrate to maximize trapping of ^89^Zr on the resin. The dissolution vial was rinsed with 2 M HCl (2 mL) and passed through the hydroxamate resin again at 1 mL/min flowrate. The ^89^Zr remained trapped on the resin as indicated by the in-process monitoring of the radiation detector at the column. The resin was washed with 2 M HCl (14 mL) at 3 mL/min flowrate followed by water (10 mL) at 3 mL/min flowrate. Finally, the ^89^Zr was eluted with 1 M oxalic acid solution (1.5 mL) at 0.5 mL/min flow rate and collected in a sterile 5 mL product vial. 

### 2.5. [^89^Zr]Zirconium-Oxalate Characterization

#### 2.5.1. Determination of Radionuclidic Purity

The radionuclidic purity of ^89^Zr was determined by gamma-spectroscopy using a high purity germanium (HPGe) detector (Ortec, Oak Ridge, TN, USA). A 4-h scan of a diluted aliquot (50–80 kBq) was performed at the end of purification. To determine longer-lived contaminants, scanning was repeated after three half-lives.

#### 2.5.2. Determination of Effective Specific Activity

The effective specific activity (MBq/µmol) of ^89^Zr was determined by labeling *p-SCN-Bz*-DFO with purified [^89^Zr]zirconium oxalate. *p-SCN-Bz*-DFO was selected as it is a commonly used chelator for labeling antibodies with ^89^Zr [17,18]. Specific activity was calculated for three consecutive batches of [^89^Zr]zirconium oxalate. A stock solution of *p-SCN-Bz*-DFO in DMSO (1 mg/mL) was prepared and 15 reaction tubes were prepared by 1:2 serial dilution in ultrapure water (500 µL) to give a final *p-SCN-Bz*-DFO concentration in the range of 2.07E-02 to 1.267E-06 µmol. A solution of [^89^Zr]zirconium oxalate was neutralized with the 2 M NaCO_3_ (pH 7 ± 0.2) and added to the *p-SCN-Bz*-DFO solutions. The resulting solutions were incubated at 37 °C on a shaker at 650 RPM for one hour. After incubation, 1 µL aliquots were spotted on iTLC and analysed by using citrate buffer (100 mM, pH 5.0) as a mobile phase. iTLC plates were measured using radio-TLC scanner. The results of the *p-SCN-Bz*-DFO titration with ^89^Zr were plotted and fitted with a sigmoidal dose-response curve to generate an EC_50_ value which was used to calculate specific activity in MBq/µmol. 

#### 2.5.3. Characterization of Radioactive Impurities Produced during Irradiation

Typical impurities in the production of ^89^Zr are ^88^Y, ^88^Zr, ^89^Y, ^90^Zr and ^56^Fe. Usually, impurities are derived from impure starting materials (^89^Y), produced during proton bombardment due to inappropriate beam energy (^88^Y and ^88^Zr) or introduced via the buffers and recourses (^90^Zr and ^56^Fe). These impurities can be reduced using proper buffers and recourses and some of these impurities were removed by purifying the ^89^Zr-solution using a hydroxamate column. All impurities were characterized using gamma-spectroscopy using a high purity germanium (HPGe) detector (Ortec, Oak Ridge, TN, USA). 

#### 2.5.4. Elemental Analysis by ICP-MS

The ICP-MS of three batches of final ^89^Zr solution to determine metallic impurities were performed and validated by an ISO 9001:2015 certified Institution (Saskatchewan Research Council, Saskatoon SK, country). Niobium and yttrium were analyzed using an Agilent 7900 ICP-MS (Santa Clara, CA, USA) and all other elemental analysis were done using an Agilent 8800 ICP-MS (Santa Clara, CA, USA). 

#### 2.5.5. Preparation of p-SCN-Bz-DFO Conjugated Antibody

Conjugation of *p-SCN-Bz*-DFO to trastuzumab (DFO-trastuzumab) was performed following published procedures with slight modification [3,19]. Briefly, trastuzumab (5 mg/mL) in PBS was buffer exchanged in 0.1 M NaHCO_3_ (pH 9) using centrifugal filters and concentrated to 10 mg/mL trastuzumab in the bicarbonate solution. A sixteen-fold mole excess of *p-SCN-Bz*-DFO (16 µg) in DMSO was added dropwise to the trastuzumab (2 mg) solution. The reaction mixture was incubated at 37 °C on a shaker at 650 RPM for an hour. The reaction mixture was cooled to room temperature and the unreacted DFO was removed by centrifugations using a spin-cap column (size 10-12 kDa). The buffer was exchanged with PBS using the same centrifugal filters. 

#### 2.5.6. Radiolabeling of DFO-Trastuzumab and Determination of Specific activity of [^89^Zr]Zirconium Oxalate

Zirconium-89 in 1 M oxalic acid was neutralized by diluting with 1 M HEPES pH 7.4 followed by adding 2 M NaCO_3_ (pH 11) dropwise until the solution was neutralized (pH 7 ± 0.2). Radiolabeling was performed using 15 different concentrations of DFO-trastuzumab and was prepared by 1:2 serial dilution in HEPES (100 µL) to give final DFO-trastuzumab masses in the range of 200 µg to 0.024 µg. Approximately 3.3 MBq of the neutralized [^89^Zr] solution was added to each reaction tube. The reaction mixture was incubated at 37 °C in a shaker at 650 RPM for two hours. The reaction mixture was cooled to room temperature and 1 µL aliquots were analysed using iTLC with 0.15 M sodium citrate as a mobile phase. iTLC was measured using radioTLC scanner. The results of DFO-trastuzumab titration were plotted and fit with a sigmoidal dose–response curve to create an EC_50_ value and used to calculate specific activity in MBq/ug.

## 3. Results and Discussion

### 3.1. Cyclotron Irradiation of ^89^Y

Zirconium-89 was produced by proton irradiation of commercially available ^89^Y sputtered on a niobium coin using the ^89^Y(p, n)^89^Zr reaction on a TR-24 cyclotron. Proton irradiation on ^89^Y-targets has been investigated by several research groups [1,12,15,20,21]. Damage to the yttrium coins (yttrium solid and sputtered coins) was reported by Queern et al. [20]. In their production method, cyclotron irradiations were performed using proton beam energy 17.8 MeV degraded to 12.8 MeV using a 0.75 mm aluminium degrader. Beam currents of 40–45 µA were applied. Using the same irradiation conditions, we did not observe any damage to the yttrium target when irradiated for up to 4 h. The activity of the purified ^89^Zr solutions was in the range of 866–1160 MBq after 2 h of irradiation using 45 µA beam current. Contamination of ^88^Zr and ^88^Y was not observed, as the formation of ^88^Zr and ^88^Y occurs via the ^89^Y(p,2n)^88^Zr reaction at higher incident proton energies >13 MeV.

### 3.2. Automation

With the vast development of ^89^Zr radiolabeled antibodies for diagnostic application using PET imaging and increasing demand for their clinical use, there is a need for cGMP grade ^89^Zr. In this study, we have adopted a commercially available cGMP-compliant TRASIS miniAiO to achieve efficient and reproducible batches of ^89^Zr. This automated module was used for the target dissolution and separation of ^89^Zr from the yttrium. In our method, the separation time including dissolution of the target was completed within 1 h. In addition, automated data acquisition was employed, allowing for full GMP-compliant documentation of the process. Furthermore, hydroxamate resin cartridge preconditioning and sterile-filter integrity testing were programmed in the same sequence file. The ^89^Zr was eluted with a very small volume of oxalic acid (1.5 mL) directly over the sterile filter into the final vial. The automation of ^89^Zr resulted in a significant reduction in the radiation dose and increased the GMP compliance.

Wooten et al. [14]. described an in-house automation system for ^89^Zr processing constructed from extruded Al modular framing attached to walls made of ultra-high molecular weight polyethylene with components mounted on the plastic walls and a computer-controlled separation. This in-house automation method suffered from rather low and inconsistent recoveries (44–97%; average 74% ± 16%), while this work had a consistent recovery of 88.2–98.1% (average 93.6% ± 5%). Unlike the in-house module, the use of the GMP-certified AllinOne ASU produced under ISO 9001 offers an easier pathway to obtaining ^89^Zr that meets cGMP needs for clinical studies. 

### 3.3. Specific Activity and Radionuclidic Purity

The specific activity in the ^89^Zr-immuno-PET imaging is vitally important because the amount of antibody injected in the preclinical or clinical subjects can alter the quality immuno-PET images and quantification. Particularly, tumour-specific antibodies or peptides for diagnostic and therapeutic applications where only small amounts of antibodies are injected to ensure site-specific uptake. Metallic impurities can compete with the radioisotope for binding sites or ligands during the radiolabelling process with a resulting decrease in radiolabelling efficiency. In order to achieve higher radiolabelling efficiency, radionuclides need to be free from metallic impurities and this can be achieved by selecting high purity target material, energy window for irradiation and trace metal grade solutions for processing. The conventional method to determine effective specific activity (ESA) of ^89^Zr is titration with commonly used chelator deferoxamine (DFO). In this method, the known quantities of *p-SCN-Bz*-DFO is titrated with ^89^Zr. The specific activity of three production batches was calculated and was found to be in the range of 1351–2323 MBq/µmol. Our effective specific activities by titration method were low compared to reported data by Queern et al. [20], but similar to those of Wooten et al. [14]. Our ICP-MS analysis showed significantly lower concentration of metallic impurities compared with those of Queern et al. [20]. 

**Figure 3:**
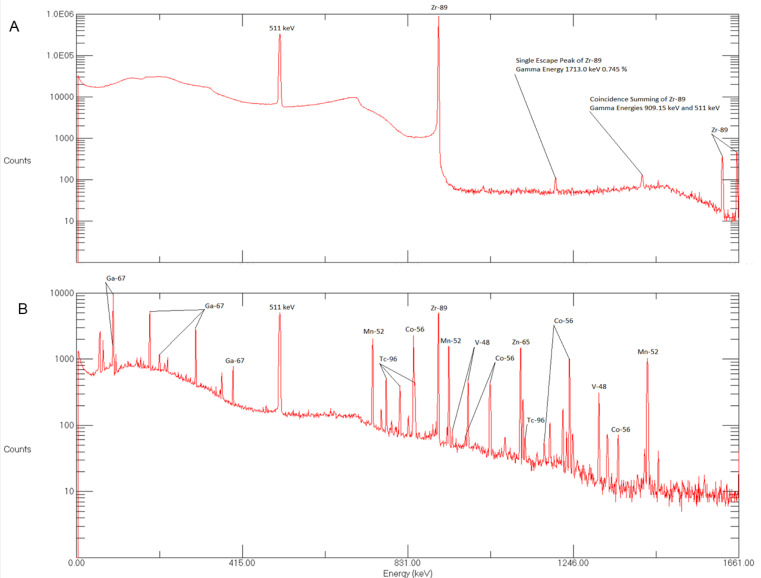
(**A**) Gamma spectrum of purified sample of ^89^Zr taken 8h after the end-of-bombardment (EOB). (**B**) Gamma spectrum of impurities found in waste vial following ^89^Zr production.

The radionuclidic purity of ^89^Zr mainly depends on the purity of the target material and irradiation conditions. The [^89^Zr]zirconium oxalate solution was tested for radionuclidic identity and purity using a high purity germanium (HPGe) detector (Figure 3A). The analysis showed the presence of 909 and 511 keV peaks. No additional radioactive contamination was detected. In these studies, the radionuclidic purity of the isolated ^89^Zr fractions was found to be >99.99%. Similar values for radionuclidic purity have been reported by other authors [1,14,20].

Gamma spectrometry of waste was also performed to determine radioactive impurities produced during irradiation. These impurities were successfully removed using hydroxamate resin as these impurities were not adhered on hydroxamate resin cartridge. The radioactive impurities produced during production were ^52^Mn, ^54^Mn, ^56^Co, ^65^Zn, ^67^Ga, ^96^Tc, ^48^V (Figure 3B).

### 3.4. Radiolabeling and Characterization of ^89^Zr-DFO-Trastuzumab

**Figure 4:**
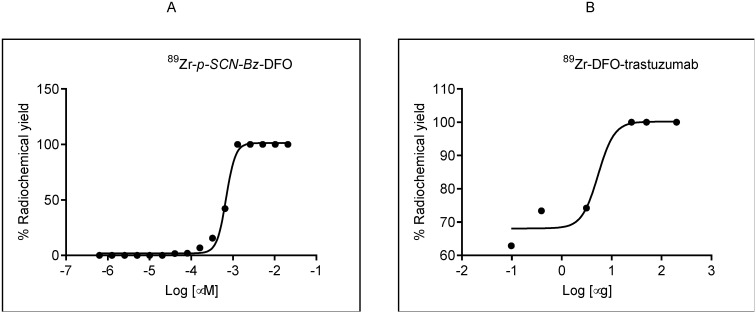
(**A**). Sigmoidal dose–response curve of *p*-isothiocyanatobenzyl-desferoxamine (*p-SCN-Bz*-DFO)–^89^Zr titration, showing effective specific activity of 2323 MBq/µmol. (**B**) Sigmoidal dose–response curve of ^89^Zr-DFO-trastuzumab titration showing effective specific activity of 0.308 MBq/µg.

Radiolabeling experiments were performed as described in the literature [3,19]. To obtain the quantitative radiolabeling yield and to determine specific activity, several reactions with different concentrations of DFO-trastuzumab were performed and analysed on iTLC (Figure 4A,B). Reported specific activities of ^89^Zr-DFO-trastuzumab typically range from 0.067 to 0.296 MBq/µg [20,22,23]. 

The labeling efficiency of ^89^Zr-DFO-trastuzumab was 100% ± 5% when 25 μg of DFO-trastuzumab and 3.3 MBq ^89^Zr was used. The specific activity was 0.308 MBq/µg. which is comparable to Queern et al. [20] (specific activity 0.296 MBq/µg with DFO-trastuzumab). The results of radiolabelling efficiencies are summarized in Table 1.

### 3.5. Elemental Analysis

The ICP-MS analyses of three batches of the final ^89^Zr solution (1.0 mL) were performed after decay of ^89^Zr. The concentrations of impurities such as yttrium, zirconium, zinc, aluminium, copper, nickel, iron, chromium, niobium and magnesium were quantified by ICP-MS (Table 2). All impurity levels were significantly lower than previously reported values [15,20] and always below <1.0 ppm

## 4. Conclusions

We report a simplified and efficient automated purification method for [^89^Zr]zirconium oxalate with high radionuclidic purity using the commercially available TRASIS miniAiO automation module. This method showed reproducible results for the production of [^89^Zr]zirconium oxalate. Additionally, the automated process with disposable cassettes provides sterile [^89^Zr]zirconium oxalate and documentation of the manufacturing process that can be used to fulfil cGMP requirements. This automated process can also be adopted for [^89^Zr]Cl_2_ production. With adequate shielding, the module ensures safe user operation and environmental radiation protection. This study strongly reinforces the utility of commercially available automated modules for the production of cGMP grade ^89^Zr.

## Figures and Tables

**Table 1:** The labelling efficiency of ^89^Zr with DFO conjugated trastuzumab were evaluated at 37 °C with varying concentrations of conjugate in HEPES buffer, pH 7. A constant ~3.3 MBq of ^89^Zr-oxalate was added to the DFO-trastuzumab solution for labeling.

Mass of DFO-Trastuzumab (µg)	Labeling Efficiency (%)
200	100
50	100
25	100
3.125	74.24
0.39	73.41
0.048	62.43

**Table 2:** ICP-MS analysis of three consecutive ^89^Zr production runs.

Sample #	Zr (ppm)	Al (ppm)	Y (ppm)	Fe (ppm)	Cu (ppm)	Cr (ppm)	Ni (ppm)	Zn (ppm)	Mg (ppm)	Nb (ppm)
PV1 ZR8920180905	0.006	0.019	0.001	0.0084	0.0003	0.0018	0.0013	0.0099	<0.1	0.045
PV2 ZR8920180907	0.006	0.014	<0.001	0.0044	0.0005	0.0016	0.0024	0.019	<0.1	0.022
PV3 ZR8920180910	0.007	0.016	0.009	0.0042	0.0004	0.0009	0.0007	0.0094	<0.1	0.018
